# Redox metals homeostasis in multiple sclerosis and amyotrophic lateral sclerosis: a review

**DOI:** 10.1038/s41419-018-0379-2

**Published:** 2018-03-01

**Authors:** Sahar Sheykhansari, Kristen Kozielski, Joachim Bill, Metin Sitti, Donato Gemmati, Paolo Zamboni, Ajay Vikram Singh

**Affiliations:** 1 Max Planck Institute for Intelligent Systems, Heisenbergstr. 3, Stuttgart, 70569 Germany; 2 Institute for Materials Science, University of Stuttgart, Heisenbergstr. 3, Stuttgart, 70569 Germany; 3 Hemostasis & Thrombosis Center - Azienda Ospedaliera-Universitaria di Ferrara, Ferrara, Italy; 4 Translational Surgery Unit, Azienda Ospedaliera Universitaria di Ferrara, via Aldo Moro 8, 44124 Ferrara, Italy

## Abstract

The effect of redox metals such as iron and copper on multiple sclerosis and amyotrophic lateral sclerosis has been intensively studied. However, the origin of these disorders remains uncertain. This review article critically describes the physiology of redox metals that produce oxidative stress, which in turn leads to cascades of immunomodulatory alteration of neurons in multiple sclerosis and amyotrophic lateral sclerosis. Iron and copper overload has been well established in motor neurons of these diseases’ lesions. On the other hand, the role of other metals like cadmium participating indirectly in the redox cascade of neurobiological mechanism is less studied. In the second part of this review, we focus on this less conspicuous correlation between cadmium as an inactive-redox metal and multiple sclerosis and amyotrophic lateral sclerosis, providing novel treatment modalities and approaches as future prospects.

## Facts

- Essential metals (e.g., iron and copper) regulate gene expression, maintain cell structure, conduct neurotransmission, and are involved in homeostasis of antioxidant response.
- Transmembrane proteins, including Ctr 1, DMT1, ATPases (ATP7A and ATP7B) play crucial roles in the intracellular copper regulation related to ALS pathophysiology.
- Cadmium is known to influence multiple sclerosis-related motor speed, attention, memory and its turnover influences Polyneuropathy.

## Open questions

- What is the correlation between metalomics and MS/ALS?
- What is the impact of crosstalk between molecular machinery regulating lesser known trace reactive metals into the MS/ALS metalomics profile?
- How does CCSVI-related extra-cranial venous strictures in MS patients influence the homeostasis of the reactive metal *via* local related arterial and venous circulation?

## Introduction

Multiple sclerosis (MS) is a demyelinating disease of the central nervous system (CNS). Its resultant inflammation causes oligodendrocyte degeneration, myelin sheath destruction, and neurodegeneration^1–4^. Although the origin of MS is still unknown, genetic predisposition and environmental toxicity activate the immune system against neural cells^5,6^. The onset of MS is often in young people aged between 20 and 40 years^7,8^. Caucasians race, particularly those of northern European descent and white women living in cold and humid areas are more affected by MS^9^.

Amyotrophic lateral sclerosis (ALS) is an adult onset, fatal, and quick destructive CNS disease, one of the most common neurodegenerative disorders with incidence around 1/100,000 with growing population in many countries^10,11^. 90% of ALS cases are sporadic and the rest are linked to genetics (familial), but their manifestations and pathological mechanisms are similar. ALS is clinically categorized as a heterogeneous disease, where the onset age, area and initial of symptoms, and speed of progression are varied among patients. Upper and lower motor neurons in the brain stem, cerebral cortex, and spinal cord are the most regions attacked by ALS. Considering clinical heterogeneity, ALS often manifests with progressive muscles atrophy, causing paralysis and death in 2–5 years after symptom onset in most patients. Respiratory failure resulted from neuronal and skeletal injury is typically identified as the primary cause of death. Despite the unknown and complex origin of ALS, numerous reasons, including redox metals dys-homeostasis, overproduced oxidative stress, mitochondrial dysfunction, neuro-inflammation, and glutamate excitotoxicity are responsible for motor neuron loss^10–13^.

**Fig. 1:**
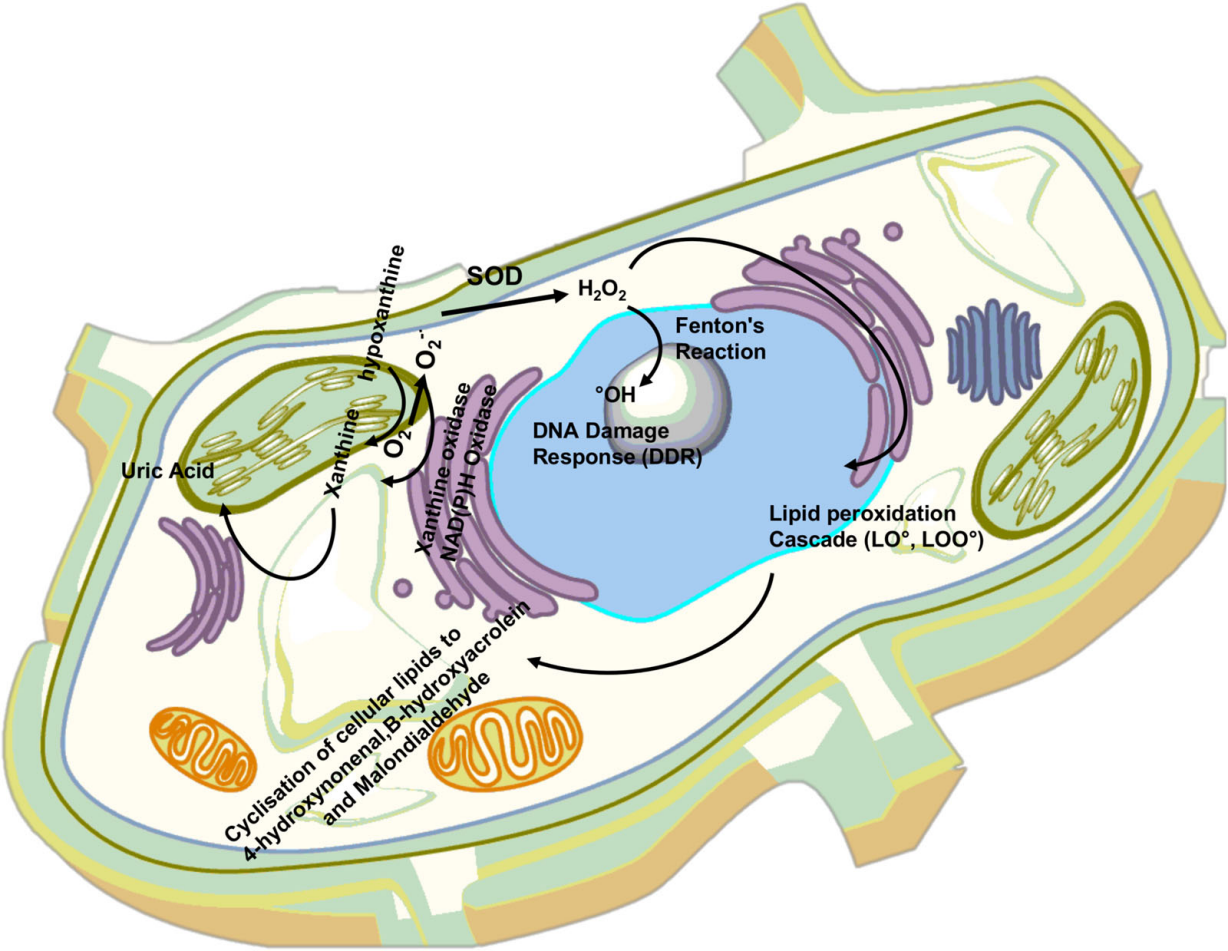
ROS and anti-ROS cellular machinery involved intracellular homeostasis of protein/lipid/DNA . ROS is formed by complex I and III in the electron transport chain in the inner layer of mitochondria through oxidative phosphorylation process, consuming oxidation of NADH or FADH to generate potential energy for protons^19,211,212^.

Metals are essential cofactors for enzymes and structural elements for stabilizing static biomolecules^14^. They also participate in principal biological metabolisms of the brain, including neurotransmitter synthesis, nerve transition, and oxygen transport^15^. Recently, notable attention has been paid to redox-active metals such as iron (Fe) and copper (Cu), and redox-inactive metals like cadmium (Cd) due to their inevitable capacity in neurodegeneration. Oxidative stress (overproduction of reactive oxygen species (ROS) and reactive nitrogen species (RNS)) is produced by either metals dys-homeostasis or an imbalance between the formation of free radicals and their destruction by antioxidants, leading to cellular damage, aging, and apoptosis through oxidation of principal cellular components (i.e., lipids, proteins, and DNA). It is obscure that metals interaction is initial or secondary factors, or outcome of the neurodegeneration^11,15–19^. Mitochondria are the main site of ROS production and cells apoptosis (see Fig. 1 for details). They are vulnerable to ROS and it has been confirmed that mitochondrial injury intensifies ROS and oxidative damage in MS^20,21^.

Herein, we review the role of redox-active metals (iron and copper) and redox-inactive metal (cadmium) in MS/ALS. First, we briefly explain the iron and copper homeostasis in the human body and the cell biology of these metals in MS/ALS. Second, we summarize recent progress on the role of iron and copper in MS/ALS. Third, we emphasize the effect of cadmium on these diseases. The last section provides our future perspectives and conclusions.

## Iron homeostasis

**Fig. 2:**
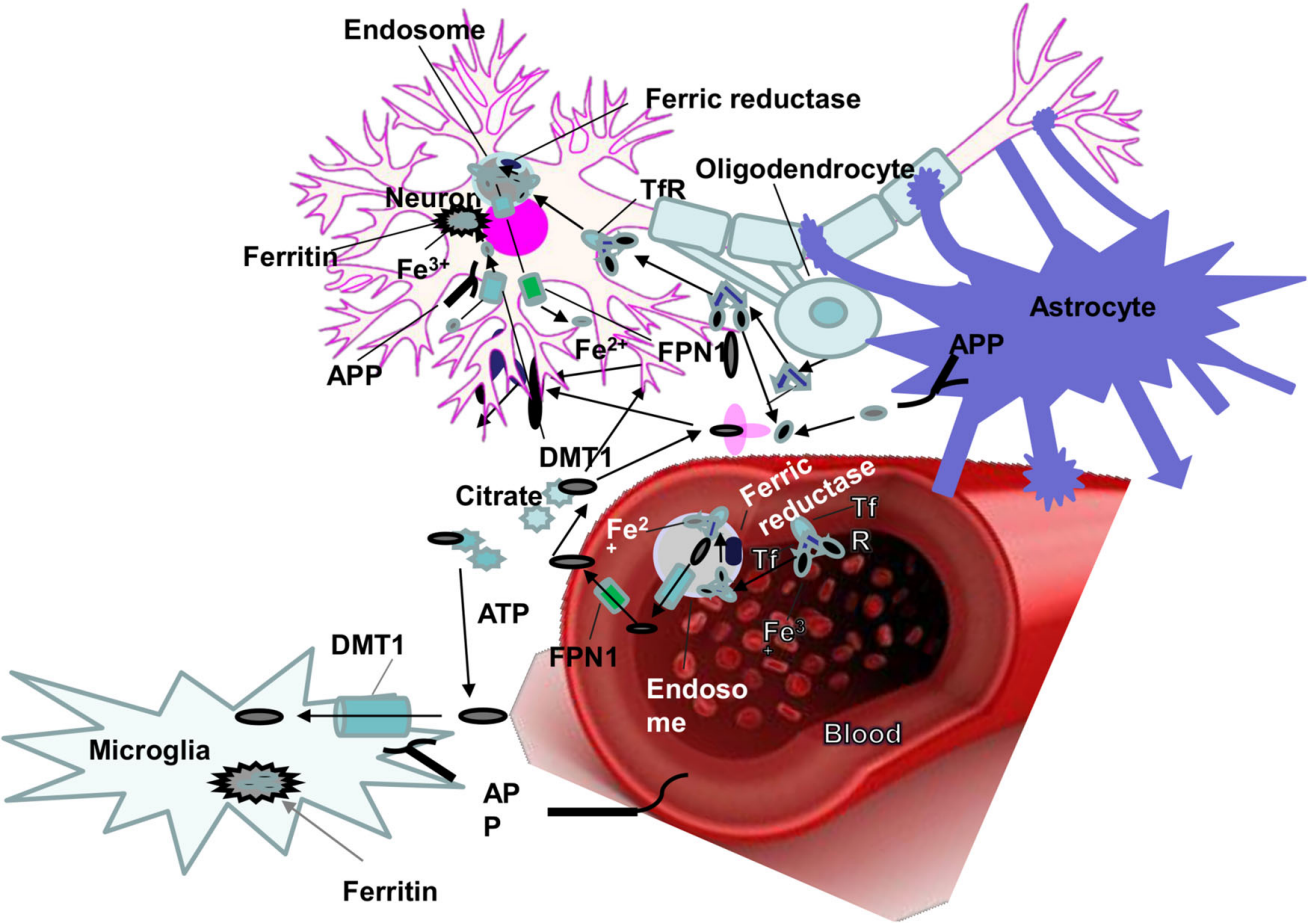
Iron metabolism in the brain. Astrocytes express CP to oxidize Fe^2+^ . Oligodendrocytes, a primary target in inflammatory attack, and synthesize Tf that controls intracellular iron transport. Microglia represent DMT1, APP, and ferritin, assisting neurons to maintain iron hemostasis in the brain environment. They also protect normal neuron function by iron regulation. The ferric iron (Fe^3+^) derived from diet, excreted enterocytes, and reticulocytes binds to transferrin (Tf). This combination uptake in the endothelial surfaces in the BBB is mediated by TfR. Fe^3+^ releases from Tf-TFR complex in the endosome and is catalyzed to ferrous iron (Fe^2+^). Thereby, TfR is recycled to bind to the iron Tf complex in the plasma. Alternatively, Fe^2+^ is transported to cytosol of endothelial cells and extracellular fluid by DMT1 and FPN1, respectively. In addition, released Fe^2+^ is quickly converted to Fe^3+^ by CP, expressed by both astrocytes and endothelial cells followed by bonding to Tf or low molecular weight molecules (e.g., citrate and ATP). Non-Tf-bound iron (NTBI) synthesized in the cytosol is the iron source for oligodendrocytes and astrocytes where Tf is highly saturated by iron^22,23^

**Fig. 3:**
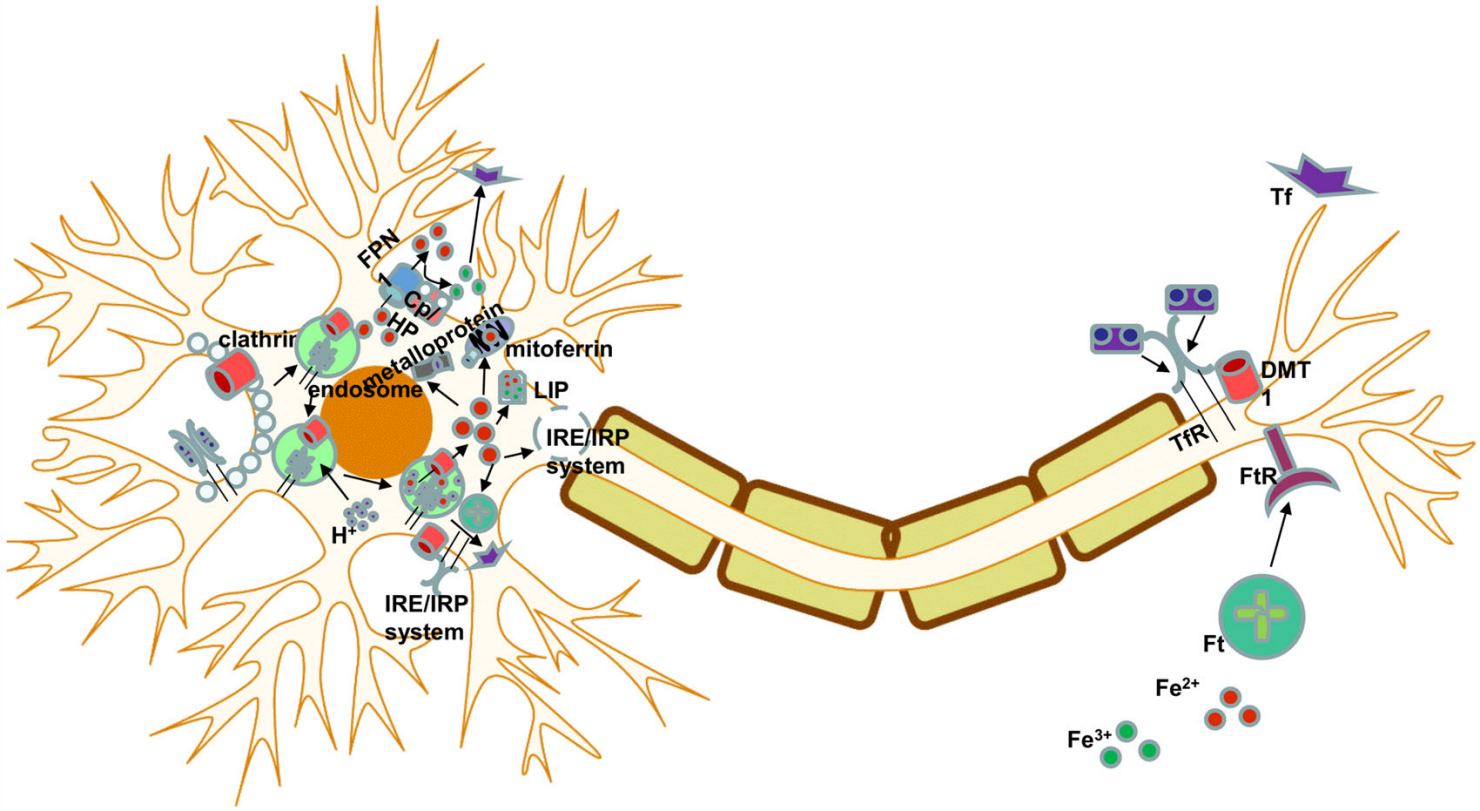
Neuronal iron homeostasis

Iron is a redox-active metal circulating between Fe^2+^ and Fe^3+^ ionic states. Cellular iron homeostasis is tuned by iron-responsive/regulatory element proteins (IRE and IRP), adjusting the process of iron uptake and storage to maintain iron balance in different cells^17^. Brain cells synthesize several receptors (*e.g*., transferrin receptor (TfR), divalent metal transporter 1 (DMT1), amyloid precursor protein (APP), ferroportin 1 (FPN1), ceruloplasmin (CP), and ferritin) and handle iron trafficking by many ways depending on functions and requirements (see Figs. 2 and 3 for details)^22,23^.

## Cell biology of Iron and its role in MS/ALS

Iron is accommodated in different parts of the human body. Sixty-five percent of iron exists in hemoglobin, 25% of the total iron is bound to storage proteins (ferritin and hemosiderin), and 10% of the iron concentration participates in the structure of myoglobin, cytochromes, and enzymes. Only 0.1% of iron binds to Tf and circulates in plasma^17,24^. In the brain, the majority of iron components is stored as non-heme iron in oligodendrocytes and myelin. The accumulation of iron is elevated by increase in age in the normal human brain, particularly after 40–50, which is the time of onset of two forms of MS known as primary progressive MS (PPMS) and secondary progressive MS (SPMS)^1^. Basal ganglia are known as the high iron content region in the brain^25^, and the elevation of iron deposition in basal ganglia is related to the normal aging process^26^.

Iron is a cofactor in the catalytic center of various enzymes for normal brain metabolism, including oxidative phosphorylation, myelination, neurotransmitter formation, etc. Moreover, iron participates in normal physiological processes within oligodendrocytes for construction of myelin where enzymes machinery utilizes iron^1^. Tight homeostasis of cellular iron is required to maintain the normal level of iron, since its excessive concentration can become deleterious for cells function^27–30^. Iron mismanagement can cause microglia activation, induction of mitochondria dysfunction, generation of free radicals in the brain^22,31–34^. Indeed, the redox capacity of free iron to carry out one-electron reactions, catalyzing the formation of ROS, is proposed as the key factor in MS/ALS^1^. It remains unclear that iron deposition is an epiphenomenon or an initializer in the MS development^32^.

Superoxide dismutase (SOD1) mutation gene is the most frequent mutation in ALS^35^, detected in 20% of familial cases and in about 2% of cases overall. SOD1 mutation participates in pathogenesis of ALS through generation of oxidative stress, cytoskeletal abnormality, glutamate toxicity, mitochondrial dysfunction, and extracellular toxicity. It is unclear whether an altered enzyme activity or indirectly a disturbance in transition metal homeostasis is involved in the disease pathogenesis^36,37^. Under the normal condition, mitochondria convert 1–3% of oxygen molecules to superoxide radicals, which are later removed by SOD1. In the absence of SOD1, slow dysmutation process causes oxidative stress. In fact, superoxide radicals release iron from iron-containing proteins (ferritin) in vivo stress conditions. They also partake in Haber–Weiss reaction, the combination of Fenton reaction and the reduction of Fe^3+^ by superoxide and formation of Fe^2+^. The liberated iron, Fe^2+^, participates in a Fenton reaction and produces free radicals (e.g., reactive hydroxyl radicals) that damage cells^24,36^.

Anomalous iron handling may be exacerbated by genetic predisposition due to particular gene variants in different iron homeostasis genes^27,38–41^. The mutation of Hfe is proposed as a risk factor of MS^40^ and ALS^36,42–44^. It leads to Hemochromatosis and decreases the expression of Cu/Zn SOD1^36,42–44^.

### Iron and MS

As mentioned above, iron dys-homeostasis can lead to neurodegeneration, which is relevant to MS pathology. The relationship between abnormal iron content and an abundance of oxidative damage is reported in MS^1,45–47^. Hametner et al. observed the elevation of iron accumulation with age increase in the white matter (WM)^1^. Iron-rich oligodendrocytes, myelin, and microglia were also destroyed in the active MS lesion, increasing the liberated iron, thereby intensifying oxidative damage and neurodegeneration^4,48,49^. Conversely, considerable reduction of iron and elevation of oxidative stress are found in MS patients^16,50^.

Numerous experimental animal models have been employed to investigate mechanisms of MS pathology. Experimental allergic encephalomyelitis (EAE) is one of the most commonly used models. Iron accumulation, which presumably comes from myelin, oligodendrocytes, and the breakdown of the blood–brain barrier (BBB), was found during active and recovery phases of EAE^47^.

Magnetic Resonance Imaging (MRI) phase have widely been used to illustrate the iron accumulation in MS lesions. Moreover, increased local field in basal ganglia can be interpreted as pathological iron accumulation and peripheral phase rings display iron-positive macrophages at the edge of lesions^51,52^. Mehta et al. showed the distribution of iron in non-phagocytosing microglia at the periphery of demyelinated sections with Perls’ stain and immunohistochemistry^53^. Iron-containing macrophages represented markers of proinflammatory (M1) contrast. Likewise, iron was preferentially taken up by non-phagocytosing, M1-polarized macrophages, and induced M1 (super) polarization in human macrophage cultures. Bagnato et al. also observed oligodendrocytes in normal WM and microglia at the edges of WM lesions as iron origins in gradient echo MRI^54^. In another example, susceptibility weighted imaging-filtered phase images demonstrated high iron content in phase MS lesions^55^. Interestingly, Adams et al. found additional source of iron in the CNS and showed that chronic inflammation and vein walls damage lead to the deposition of hemosiderin within and outside of lesions^56^. The histologic evidence of red blood cells extravasation through the BBB is currently mirrored by the frequent MRI of micro-bleedings around the brain venules^54,56,57^. Moreover, differently from the other neurodegenerative disorders, each MS lesion is crossed by a central venule, which may exhibit increased pressure as a consequence of restricted outflow in the jugular system. Therefore, chronic cerebrospinal venous insufficiency (CCSVI) may favorite the BBB leakage and iron loading of heme origin^58^.

Iron deposition can be represented by T_2_ hypointensity or black T_2_ in MRI. Gray matter (GM) T_2_ hypointensity in MS patients is related to physical disability, brain atrophy, and disease course^59,60^. Bakshi et al. reported black T_2_ in subcortical nuclei in wheelchair-bound and SPMS patients prominently correlated to longer disease course and ambulatory disorders^59^. Additionally, the role of iron is emphasized in cognitive disorders of MS patients^61,62^. A study by Brass et al. revealed the relationship between T_2_ hypointensity, cognitive impairments, and the effect of iron content in basal ganglia in neuropsychological disorders^61^.

The demyelination of WM has long been considered as a significant sign of MS, though, the importance of the GM demyelination is emphasized^63,64^. Indeed, high iron deposition in deep GM is shown in MS cases^34,65,66^. It has been proposed that WM damage disturbs the axonal iron transition and elevates the iron deposition in deep GM^34^. Haider et al. suggested the correlation between high iron bulk, deep GM demyelination, and clinical disability^65^.

### Iron and ALS

Since iron is known to promote the motor neuron degeneration, some trials have examined the iron state in ALS patients. It has been demonstrated that the excess ferritin level can worsen muscle degeneration and shorten patients’ survival^10,67–69^. High iron concentrations is also reported in the spinal cord of ALS patients^70–73^. Hozumi et al. reported an increase of the iron content in the cerebrospinal fluid of ALS cases^74^. Interestingly, Mizuno et al. noticed the presence of Tf (an iron regulator) in Bunina bodies and some of basophilic inclusions, where the pathogenesis of ALS occurs^75^.

Similar to MS, T2 shortening reveals iron deposition in the brains of ALS patients and iron is considered as a biomarker for ALS^76–78^. Nevertheless, Hecht et al. declared that hypointensities are not due to iron accumulation in ALS patients and offered alternative sources like free oxygen radicals^79^.

Animal models have been adopted to demonstrate the association between iron and ALS. The disturbance of iron homeostasis was observed in a murine model and it has since been suggested that the inhibition of axonal transport iron, perturbation of proteins adjusting the iron influx, and elevation of mitochondrial iron storage in neurons and glia lead to iron accumulation in SOD1 transgenic mice^37^. The blood accumulation in damaged blood vessels and iron deposition created motor neuron degeneration in SOD1 transgenic mice^80^. .Also, the elevation of iron-related mRNA expression increased iron and subsequent oxidative damage in SOD1-G93A mice^12^. In some trials, iron chelator therapy has been administered in a G93A-SOD1 murine model of ALS, resulting in neuroprotective effects and increased lifespan^37,81,82^.

## Copper homeostasis

**Fig. 4:**
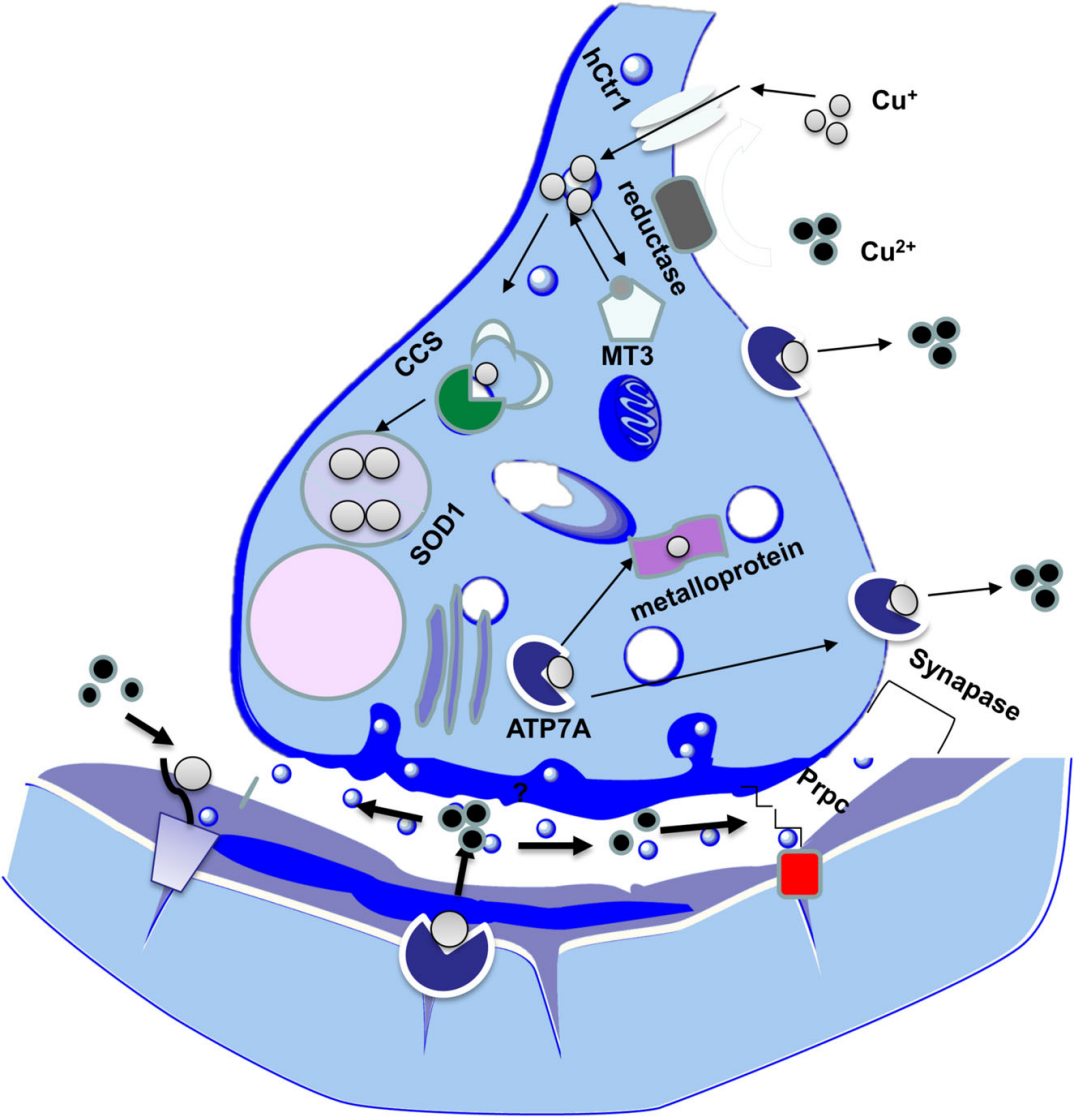
Copper homeostasis in the brain. A group of transmembrane proteins including Ctr 1, DMT1, ATPases (ATP7A and ATP7B) play crucial roles for intracellular copper regulation. Ctr1 is an essential copper transporter expressed in intestinal and brain cells to handle copper influx. DMT1 is also expressed in brain tissues and may contribute to copper uptake^86,88^. ATP7a acts as a critical source of brain copper and mediates copper movement across the basolateral membrane into the extra-vascular space of the brain. It also exports copper for subsequent incorporation into Cu-dependent enzymes^15,86,96^. ATP7b also transfers copper across membranes, however, the function of ATP7b is less clear compared to ATP7a^86,88^. Copper chaperone proteins control copper traffic and delivery into specific cellular targets. Moreover, chaperone for SOD1 (CCS), chaperone for cytochrome C oxygenase (Cox17), anti-oxidant protein 1 (Atox 1) deliver copper to SOD1, cytochrome oxidase, and ATP7a, respectively^15,86,88^. MTs are low molecular weight proteins with neuroprotective roles and a high number of cysteine residues for metal binding such as copper and zinc^86,88,154^. There are four types of MTs in mammals. MT1 and MT2 are expressed in all tissues, MT3 exists in CNS, and MT4 is found in the stratified squamous epithelia. MTs are known as copper buffers in the glutamatergic synapse where excess copper induces a high level of MTs^86,88^.

The majority of dietary copper is absorbed by the small intestine and stored in the liver. The biliary pathway is responsible for 80% of copper excretion through the liver^24^. In the blood stream, around 65–90% of serum copper is attached to Ceruloplasmin, while remaining is bound to serum albumin, transcurein, and amino acids for delivery to tissues^24,83^. Only unbound copper ions can pass through the BBB^84^. The basal ganglia, cerebellar granular neurons, neuropil of the cerebral cortex, astrocytes, and hippocampus can accommodate high concentrations of copper^15^. Astrocytes are the primary cells regulate extracellular ions in the brain^85,86^. Moreover, astrocytes are considered as major contributors to copper homeostasis and high storage region of copper (see Fig. 4 for details)^86,87^.

## Cell biology of copper and its role in MS/ALS

Copper participates in the structure and function of several brain enzymes, regulating neurotransmitters synthesis, iron metabolism, oxidative defense, etc. Since most of the copper is presented as cuprous (Cu^+^) and cupric (Cu^2+^) ions in biological systems, it mediates electron transfer in redox reactions^88^. Therefore, cellular copper availability should be precisely adjusted for essential enzyme activity and prevention of oxidative damage^15,24,83,89–96^. The measurements showed abnormal copper level in ALS patients^97,98^. The high concentration of copper generates oxidative stress through two mechanisms, that is, catalyzing the production of hydroxyl radicals in a Fenton-like reaction as redox-active form and reducing the GSH (an antioxidant that removes ROS).

### Copper and MS

The role of copper in MS pathology is proposed to be via excessive copper and subsequent oxidative damage^9,99–102^. The injury of mitochondrial electron transport system, cytochrome oxidase, and activated glia increase copper contents^103^. However, conflicting findings have also been reported^104–111^.

Cuprizone drug administration (a copper chelator) in animals is a method to model toxic de/remyelination of the CNS. This model supports the idea that copper dys-homeostasis can mediate ROS. Furthermore, Cuprizone carries copper into the CNS and induces prominent demyelination lesions through oxidative stress and oligodendrocytes toxicity^112–123^. Animal studies have also demonstrated the positive effect of Clioquinol (CQ) as another metal chelator on suppression of different neurodegenerative diseases (i.e., Parkinson’s and Alzheimer’s)^124,125^. Choi et al. found that CQ can diminish the activation of microglia in the spinal cord of EAE and improve clinical symptoms^126^.

### Copper and ALS

SOD1 genetic mutation has been extensively investigated in animal models of ALS that express human mutant SOD1 protein. SOD1 is a major copper-binding protein with the highest affinity to copper and scavenger function of free radicals^127^. In addition to redox homeostasis, SOD1 plays a critical role on intracellular copper buffering^128^. Evidence from clinical trials confirmed that the elevation of SOD1 (D90A and G93A) levels lead to the disruption of copper metabolism and its accumulation in the spinal cord of mice, the most common region injured by ALS^129–133^. Although wild-type SOD1 is considered non-pathogenic, the overexpression of wild-type human SOD1 enhances the amount of copper in an age-related manner^130^, and causes neurotoxicity in the spinal cord^134^. Interestingly, Tokuda et al. observed high copper content in the outside SOD1 active region (non-SOD1 Cu level), which promotes disease progression in transgenic mice^130^. Regardless of SOD1 copper-binding ability, copper-regulating proteins are also affected by SOD1 mutant expression, leading to abnormal copper accumulation. Indeed, mutant SOD1 alters the expression of CTR1 and ATP7a as copper importer and exporter, respectively^129,130^. Additional trials on the spinal cord of transgenic mice revealed low copper concentration in mutant SOD1^133,135,136^, and a correlation between the degree of low copper content and ALS manifestations^137^.

There are three forms of SOD1, varying by the metal content, which are as follows: (a) fully metalated form (Holo) SOD1 that bind to one copper and one zinc, (b) demetalated (apo) SOD1, and (c) metal-deficient SOD1 that bind to one metal, copper or zinc. The fully metalated form has high stability and half-life time^138^. Enormous ALS-associated SOD1 mutations alter metal binding affinity and protein structure. Copper deficiency leads to improper hydrophobicity in wild and mutant types of SOD1, while adding copper can improve this defect^139,140^. Moreover, copper deficiency has been shown in recombinant SOD1 mutant and >50% of SOD1–G37R proteins in the spinal cord of transgenic mice^141–144^. The copper treatment could increase the concentration of the fully metalated form^144^. Nevertheless, a study claimed that a G37R mutation is inert to the SOD1 structure in its metals binding region^145^.

A trial in mice with genetically modified copper-regulating enzymes demonstrated the role of copper homeostasis in SOD1 toxicity. The CCS overexpression in SOD1-G93A mice can worsen the disease progression, though, it promotes the holo-SOD1 form^146^. This defect can be treated by copper delivery with a drug known as CuATSM^147^. Also, the deletion of CCS in SOD1 transgenic mice caused reduction of copper loaded SOD1. Interestingly, other copper-dependent enzymes and the disease progression are not affected by this procedure^148^. These suggest that copper trafficking by CCS is not related to SOD1 toxicity^149^.

Metallothioneins (MTs) are also crucial for balancing the intracellular copper content^150^, and provide copper for SOD1 and other enzymes^151,152^. The use of MTs in copper dys-homeostasis confirmed the important role of MTs in pathological signs without altering the SOD1 function^153–157^. The delivery of MT-III improved motor neurons loss^155^, whereas the reduction of MTs expression prominently enhanced disease onset and progression in SOD1 mice^156^. The diminution of MT-III mRNA was confirmed in the sporadic ALS^158^. Additionally, the effect of Dexamethasone on the elevation of MTs and decrease of the disease progression was proved in G93A-SOD1 mice^153,159^. Conversely, the elevation of astrocytic MTs immunoreactivity was observed in the spinal cord of ALS patients^160^.

Copper delivery therapy with CuATSM that releases copper into oxidative tissues could promote the survival of animal ALS models^144,147,161–163^. Utilization of CuATSM in SOD & CCS mice reduced mortality and motor neuron deficit in symptomatic cases^147^. Also, CuATSM is suggested to help familial SOD1 ALS patients, representing human CCS. On the other hand, various copper chelators, including Ammonium tetrathiomolybdate (TTM)^130,164^, d-penicillamine^165^, Trientine^166–168^, and lipophilic metal chelator were examined in SOD1 mice^169,170^. They could remove copper accumulation, inhibit peroxidase action of SOD1, and postpone disease progression^130,165–171^.

Pyrrolidine dithiocarbamate (PDTC) (an antioxidant drug) regulates proinflammatory and apoptosis genes. Despite the beneficial effect of PDTC in animals models of diseases like Alzheimer’s^172^, it decreased the survival of G93A-SOD1 ALS rat models^173^. Additionally, the copper distribution was also elevated in the spinal cord of these rats, suggesting that the excess copper may increase neurotoxicity of mutant SOD1. Similarly, oral administration of PDTC enhanced the copper concentration and the level of lipid peroxidation products due to the oxidative stress in the rat peripheral nerve^174^.

## Cadmium

**Fig. 5:**
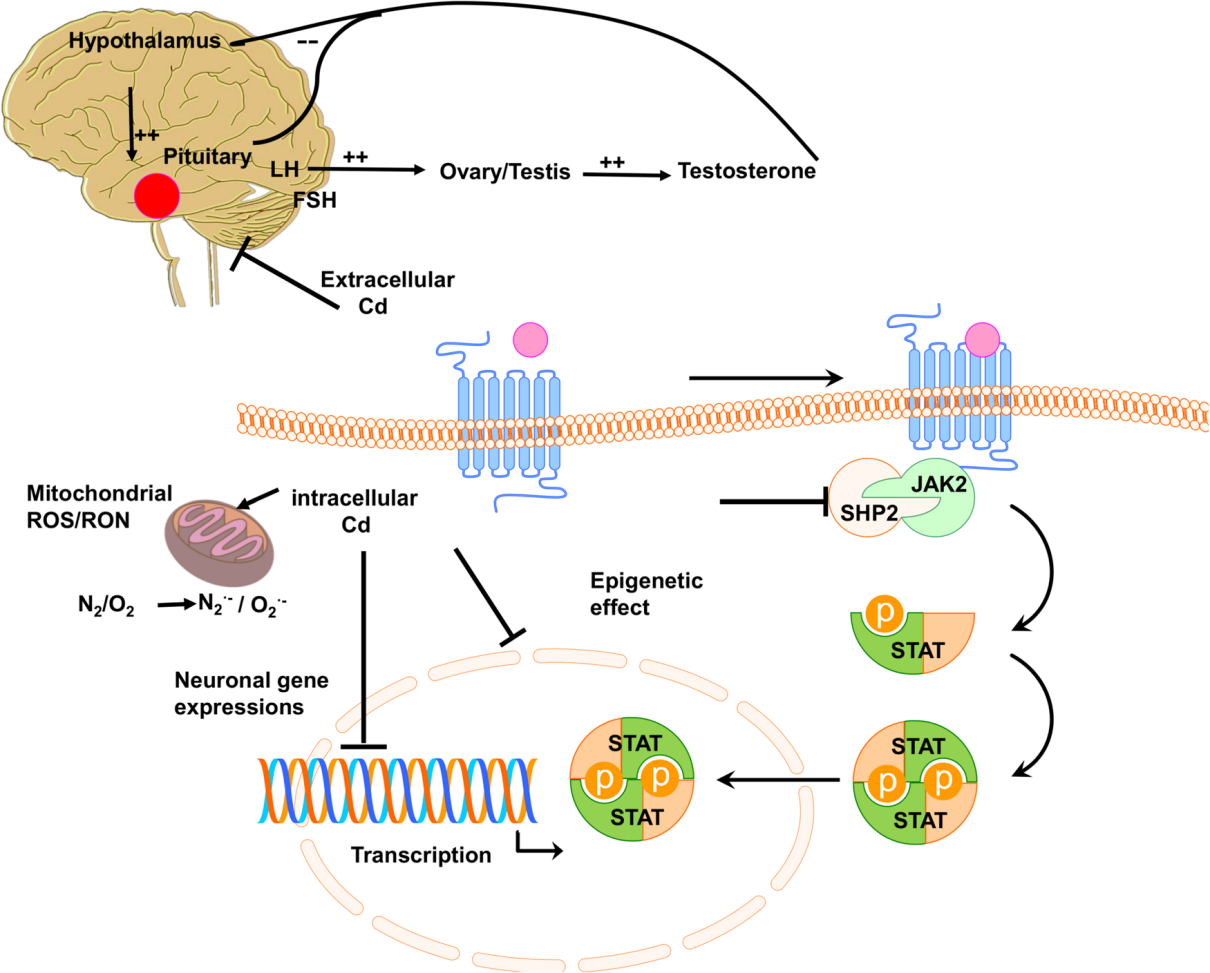
Cadmium influence on intra- and extracellular neuronal homeostasis, contributing to CNS pathophysiology. Extracellular cadmium has an estrogen-like effect, disturbing hormonal balance *via* the hypothalamic-pituitary-gonadal pathway. Intracellular cadmium disturbs neurogenesis and leads to neuronal apoptosis and ROS by impairing mitochondria signaling and inhibition of Jak/Stat signaling. The cadmium accumulation in the brain alters gene expression and causes epigenetic effects through DNA binding^175^. Moreover, it leads to oxidative stress *via* inhibition of antioxidant enzymes, depletion of antioxidants, dislocation of redox active metals, and suppression of the mitochondrial electron transport chain^17,212^. The replacement of iron and copper by cadmium, and thereby the increase of free iron and copper content, generate hydroxyl radicals and promote oxidative stress *via* Fenton’s reaction^6,17,24,212^. Additionally, the activity of different antioxidant enzymes, including Cu/Zn SOD1, glutathione peroxidase, glutathione reductase, and catalase is altered by cadmium intoxication^213^. Cadmium-induced selenium deficiency causes depletion of glutathione peroxidase^17^. Also, cellular antioxidant GSH is disrupted by cadmium and results in the elevation of ROS^214^. The excess of intracellular ROS inhibits the neural janus kinase (Jak) and tyrosine kinase, and leads to disruption of neural mitochondria^215^

Cadmium is categorized as a redox-inactive metal that cannot generate free radicals directly. It is rapidly absorbed by vegetables and grains, the main sources of dietary cadmium. Cadmium oxide produced during smoking is mainly responsible for cadmium exposure and deposition in lungs or other organs^17^. Cadmium may not behave like a metal ion, which participates in routine metabolism. Thus, human physiology is not evolved to handle cadmium metal tolerance and resistance (Table 1)^175^. Several decades ago, the toxic effect of cadmium on industrial workers was confirmed^176,177^. Its accumulation in tissues creates severe organ damage in the brain, kidney, lung, and testis^24^. . A combination of Cd and MT generates Cd–MT complex in the human body as reported in other divalent metal ions^6,178^. Importantly, cadmium influences the intra–extra neuronal homeostasis (see Fig. 5 for details).

**Table 1:** Literature review on Cd neurotoxicity in humans and rats

Year	Study design	Age group	E/C (*n*)	Exposure to Cd	Expose pathways	Effects	References
1961	Cross-sectional	Male worker	106E/84C	—	Occupational exposure	Anosmia	^216^
1977	Cross-sectional	Children	31E/22C	CdH	Daily life	Neurological disorders, such as learning disabilities and hyperactivity	^217^
1981	Cross-sectional	Children	73E/44C	CdH	Daily life	Dyslexic, learning disorder	^218^
1981	Cross-sectional	Workers	49E	CdU	Occupational exposure	Polyneuropathy	^219^
1982	Cross-sectional	Children	149	CdH	Daily life	Effect on verbal I.Q	^220^
1985	Case-control	Young men	40	CdH	Daily life	Behavioral difficulty	^221^
1985	Cross-sectional	Children	69	CdH	Daily life	Nonadaptive classroom behavior, affected behavioral development visuomotor skills ↓	^222^
1989	Cross-sectional	Male workers	31E	CdU	Occupational exposure	↓ Attention, memory, and psychomotor speed	^223^
1992	Cross-sectional	Worker	38E	CdU	Occupational exposure	90% headache; 42% dizzy spells 21% weakness; 16% brain atrophy	^224^
1992	Cross-sectional	Worker	55E/16C	CdU	Occupational exposure	Hyposmia	^225^
1997	Case report	Old man	1	Multiple organ failure	Occupational exposure, acute	Parkinsonism	^226^
1999	Cross-sectional	Worker	13E/19C	CdU	Occupational exposure	Polyneuropathy	^227^
2000	Cross-sectional	Adult worker	42E/47C	CdU	Occupational exposure	↓ Motor speed, attention, memory ↑ equilibrium, PNP, and concentration complaints	^228^
2006	Case report	Adult worker	1	CdU	Inhale the fumes	Peripheral neuropathy	^229^
2009	Cross-sectional	Children	549	CdH	Daily life	Withdrawal, social problems and attention problems associated	^230^
2012	Wister rats	Male	20E/20C	Intratracheal instilletion	Experiment exposure	Dose- and time-dependent shif from slower to faster waves	^231^

*E* exposed subjects, *C* control subjects; *CdU* urinary cadmium concentration; *CdH* concentration of cadmium in hair, *IQ* Intelligence Quotient

Some studies demonstrated the direct relationship between cadmium and MS/ALS. An earlier study proved that the cadmium exposure can lead to retrograde axonal transport and neurotoxicity in rat motor neurons^179^. In other reports, cadmium injected to animals produced ROS, which in turn changed membrane fluidity, intracellular calcium levels^180^, lipid peroxidation, and protein carbonylation^181^. Also, cadmium induced neurotoxicity through decrease of GSH level in hippocampus and midbrain^182^, increase of free radicals, mitochondrial membrane dysfunction, and cell apoptosis in brain tissues^183^.

### Cadmium and MS

Compared to iron and copper, few studies are available about cadmium toxicity in neurodegenerative disease like MS. Some reports showed high levels of cadmium in MS patients^5,6,184^. Aliomrani et al. found high concentration of cadmium in MS patients and its relationship with glutation-S-transferase (i.e., an oxidative stress correlated gene and metals biotransformation regulator)^184^.

Environmental factors are the key reasons for increasing the cadmium level in the human body. Excess cadmium contents are found either in MS patients living in industrial areas or in foods such as corn, rice, and wheat in industrial areas^185^. Also, the long-term exposure to air pollutions consisting of cadmium has potential roles in pathogenesis of MS^186^.

### Cadmium and ALS

Cadmium mediates neurotoxicity and motor neuron disease by reducing Cu/Zn SOD1 enzyme, disrupting the BBB, and glutamate toxicity via upregulation of glutamate dehydrogenase and downregulation of glutamate uptake in glial cells (Fig. 5). These effects support the hypothesis about the relationship between cadmium and ALS pathogenesis^97,187–190^. The high concentration of cadmium is reported in CNS, blood, WM, and GM of the brain in ALS patients^97,189,190^. Wu-Tao et al. showed disruption of motor neuron conduction, Cu/Zn SOD1 activity, and spinal motor-neurons function in the cadmium-treated rat^188^. Interestingly, symptoms of disease and lab data from electromyography in battery factory workers showed that ALS onset was due to cadmium neurotoxicity through the reduction of SOD1 activity. Also, high levels of MTs and MT-bound cadmium in the liver and kidney, a sign of exposure to heavy metals, was found in patients^187,191^. Similarly, experiment on *Escherichia coli* showed the effect of cadmium on the reduction of SOD1 activity by misfolding of Cu/Zn SOD1 protein and elevation of MTs expression^192^. Conversely, another study reported no relationship between cadmium and ALS^193^.

## Outlook and future prospects

New candidate metals (e.g., cadmium, arsenic, and nickel), which are indirectly involved in the ROS production, generally have long biological half-life due to the lack of a bodily recognition system. Therefore, these metals need extensive screening, particularly due to recent evidence of physiological correlations to cardiovascular diseases and CCSVI-MS. Recent developments in imaging techniques for metal detection may contribute significantly to elucidate the role of iron in MS/ALS pathology, and to develop metal-based disease biomarkers. Advanced MRI techniques and susceptibility weighted neuroimaging can detect metal deposition in the brain with high sensitivity^194–196^. Ultra-small superparamagnetic iron oxide (USPIO) nanoparticles can visualize pluriformity and cellular infiltration in MS precisely. Therefore, USPIO-enhanced MRI can be a new marker for observing WM inflammation, which cannot be visualized by routine techniques^197^. One important consideration is early exposure of these metals during pregnancy and childhood, and their late onset and association with MS/ALS. In this context, heritable changes without substantial DNA changes and their epigenetic correlations could shed new evidences about metal-induced biomolecular alterations in MS/ALS. Unlike mutations, epigenetic changes can be reversible and responsive to environmental influences, but can also have a profound impact on genetic expression. Formation of 5-methylcytosine through DNA methylation at the 5′ position of the cytosine ring of CpG Island to could be an epigenetic marker that regulates gene silencing/activation, as shown in few reports in response to metal exposure to biological systems^198^. ROS/RNS-mediated DNA damage can cause an imbalance in normal methyltransferase activity, leading to dysregulation. Aberrant gene expression due to gene-specific hypo/hyper-DNA methylation may lead to diminished glutathione activity, making neuronal systems prone to oxidative stress.

Although the involvement of iron dysregulation in MS seems apparent, the disease mechanism has yet to be clearly delineated. Many clinicians argue that there is inadequate evidence to support the iron-related hypothesis in MS/ALS. The limited efficacy of current therapies in the prevention of relapse/disability warrants exploration of alternative possibilities. MS patients differ widely in clinical outcome and symptoms as evident from plethora of literature, except those clinically definitive MS diagnosed patients. These uncertainties make MS disease pathogenesis worse. Integrating iron homeostatic biochemical markers with genetic screening could be used as novel MS treatment. It could tremendously help to identify MS cohorts requiring nontraditional treatment plans, to target MS pathogenesis aggravated by iron deposition in the brain. It may also help such patients to regulate their dietary iron supplement to avoid uncertainty related with iron in MS/ALS. If disease-related peripheral blood iron level and molecular regulators such as ferritin, transferrin saturation are known among such subgroup, clinical improvement can be achieved via dietary iron supplementation. Several clinical trials are underway to create a systematic approach to identify individualized therapy (Table 2). An interesting future approach to MS diagnostics could include correlating metal concentrations in blood with positive MRI-based diagnoses of MS. It could lead to earlier and more routine detection of MS. This may fill the gap created due to the lack of satisfactory treatments and will put forth a better prospect of obtaining definitive clinical evidence for efficacy/fail-safe therapeutic design. Last but not the least, the use of untethered, wirelessly controlled, mobile, milli/microrobots as unconventional approaches can be utilized to enhance the quality of life in post-treatment of MS/ALS while reducing emotional load, recovery time, and cost, which in recent times became a topic of choice among clinicians for future prospects^199–204^. Collaborative efforts between robotic researchers, clinicians, biomedical engineers, materials scientists and chemists have led to superior biomaterial-based design of daily need utilities to reduce the toxic metals exposure^199–201,206–208^. Plaques and implants could be future blockbuster therapeutic procedure to cure MS/ALS^209^.

**Table 2:** Clinical Trials in Iron and copper related with MS and ALS pathology (Data from clinicaltrials.gov)

Interventions	Study phase	Condition	Location
Drug (Rebif)	4	MS	EMD Serono, Inc.Rockland, Massachusetts, USA
Injection (autologous stem cells)	2	MS	NA
Drug (dexrazoxane plus Mitoxantrone and Placebo plus Mitoxantrone)	2	MS	Department of Neurology, St. Josef-Hospital, Ruhr-University Bochum, Germany
Drug (ferumoxytol)	Early phase 1	MS	National Institutes of Health Clinical Center, 9000 Rockville Pike, Bethesda, Maryland, USA
Drug (deferiprone and Placebo oral table)	2 and 3	ALS	NA
Drug (deferiprone)	2	ALS	Hôpital Roger Salengro, CHRU de Lille, Lille, France
Drug (copper)	2	ALS	Phoenix Neurological Associates, Phoenix, Arizona, USA
Drug (Cu(II)ATSM)	2	ALS	Macquarie University, Sydney, New South Wales, Australia

## Conclusion

We highlighted the recent progress in the role of redox metals in MS/ALS. The redox capacity of iron and copper, their contribution to Fenton reactions, and production of oxidative stress are identified as prominent factors for neurodegeneration in MS/ALS. Moreover, metals dys-homeostasis is reported to cause oxidative damage. The role of SOD1 mutation on copper dys-regulation in neurodegenerative disorders, particularly in ALS has been noticed by some researchers. In addition, metal chelator therapy in animal models of these disorders rescue neuronal degeneration and increased survival. On the other hand, there have been few studies on the effect of cadmium on MS/ALS; its neurotoxicity has proved through oxidative stress formation, reduction of antioxidant enzymes activity, and cellular antioxidants. Despite extensive studies to date, the main cause of these neurodegenerative disorders is still unknown and requires further investigation.
